# Acute kidney injury in infants hospitalized for viral bronchiolitis

**DOI:** 10.1007/s00431-023-05029-6

**Published:** 2023-05-24

**Authors:** Pierluigi Marzuillo, Anna Di Sessa, Raffaella Golino, Paola Tirelli, Maeva De Lucia, Giulio Rivetti, Emanuele Miraglia del Giudice, Stefano Guarino, Felice Nunziata

**Affiliations:** 1 Department of Woman, Child and of General and Specialized Surgery, Università degli Studi della Campania “Luigi Vanvitelli”, Via Luigi De Crecchio 2, 80138 Naples, Italy; 2 Department of Pediatrics, AORN Sant’Anna e San Sebastiano, via Ferdinando Palasciano, 81100 Caserta, Italy

**Keywords:** Viral bronchiolitis, Acute kidney injury, Respiratory syncytial virus, Small for gestational age, Preterm birth

## Abstract

We investigated prevalence of and factors associated with acute kidney injury (AKI) in a group of patients hospitalized with viral bronchiolitis. We retrospectively enrolled 139 children (mean age = 3.2 ± 2.1 months; males = 58.9%) hospitalized for viral bronchiolitis in a non-pediatric intensive care unit (PICU) setting. The Kidney Disease/Improving Global Outcomes creatinine criterion was used to diagnose AKI. We estimated basal serum creatinine by back-calculating it by Hoste (age) equation assuming that basal eGFR were the median age-based eGFR normative values. Univariate and multivariate logistic regression models were used to explore associations with AKI. Out of 139 patients, AKI was found in 15 (10.8%). AKI was found in 13 out of 74 (17.6%) patients with and in 2 out of 65 (3.1%) without respiratory syncytial virus (RSV) infection (*p* = 0.006). No patient required renal replacement therapies, while 1 out of 15 (6.7%) developed AKI stage 3, 1 (6.7%) developed AKI stage 2, and 13 (86.6%) developed AKI stage 1. Among the 15 patients with AKI, 13 (86.6%) reached the maximum AKI stage at admission, 1 (6.7%) at 48 h, and 1 (6.7%) at 96 h. At multivariate analysis, birth weight < 10th percentile (odds ratio, OR = 34.1; 95% confidence interval, CI = 3.6–329.4; *p* = 0.002), preterm birth (OR = 20.3; 95% CI = 3.1–129.5; *p* = 0.002), RSV infection (OR = 27.0; 95% CI = 2.6–279.9; *p* = 0.006), and hematocrit levels > 2 standard deviation score (SDS) (OR = 22.4; 95% CI = 2.8–183.6; *p* = 0.001) were significantly associated with AKI.

*Conclusion*: About 11% of patients hospitalized with viral bronchiolitis in a non-PICU setting develop an AKI (frequently mild in degree). Preterm birth, birth weight < 10th percentile, hematocrit levels > 2SDS, and RSV infection are significantly associated with AKI in the setting of viral bronchiolitis.

**Table:** 

**What is Known:** *• Viral bronchiolitis affects children in the first months of life and in 7.5% of cases it can be complicated by acute kidney injury (AKI).* *• No studies investigated associations with AKI in infants hospitalized for viral bronchiolitis*.	
**What is New:** *• About 11% of patients hospitalized with viral bronchiolitis can develop an AKI (frequently mild in degree).* *• Preterm birth, birth weight <10th percentile, hematocrit levels > 2 standard deviation score, and respiratory syncytial virus infection are associated with AKI development in infants with viral bronchiolitis.*

**What is Known:**

*• Viral bronchiolitis affects children in the first months of life and in 7.5% of cases it can be complicated by acute kidney injury (AKI).*

*• No studies investigated associations with AKI in infants hospitalized for viral bronchiolitis*.

**What is New:**

*• About 11% of patients hospitalized with viral bronchiolitis can develop an AKI (frequently mild in degree).*

*• Preterm birth, birth weight <10th percentile, hematocrit levels > 2 standard deviation score, and respiratory syncytial virus infection are associated with AKI development in infants with viral bronchiolitis.*

## Introduction

Viral lower respiratory tract infections have a great impact on children’s health [1]. Every year, in the USA, about 20% of the children aged < 12 months require outpatient medical visits because of respiratory syncytial virus (RSV) respiratory infection [2]. Furthermore, between 2 and 3% of children younger than 1 year of age are hospitalized for bronchiolitis, accounting for 47,000–172,000 hospitalization annually [2, 3].

In previous studies, our research group showed that acute kidney injury (AKI) can complicate several common pediatric conditions and that it can be frequently under-recognized in children, especially in case of mild forms [4–7].

In the current literature, only one study shows that 7.5% of infants with viral bronchiolitis develop AKI as complication of the respiratory disease [8]. More specifically, AKI develops in 12.7% of the infants with viral bronchiolitis admitted and in 4.5% of those not admitted to pediatric intensive care unit (PICU) [8]. Factors associated with AKI in infants affected by viral bronchiolitis, however, have never been investigated.

AKI is an evolutive process that can shift from a functional to an intrinsic form [9]. This latter is characterized by a structural damage of the kidneys and it represents the worst kidney involvement in this setting [9]. Being aware of the risks of AKI in infants affected by viral bronchiolitis can be clinically relevant in order to early diagnose and prevent the progression of AKI and even more to suggest a specific post-discharge nephrological follow-up [10]. In fact, even a mild AKI episode doubles the risk of chronic kidney disease (CKD) in the following years [11].

For these reasons, we aimed investigating the prevalence and factors associated with AKI in a group of patients hospitalized with viral bronchiolitis.

## Methods

Clinical and biochemical data of all patients consecutively discharged with the primary diagnosis of viral bronchiolitis from the Department of Pediatrics placed in the Sant’Anna e San Sebastiano Hospital, Caserta, Italy from January 1st, 2017 to December 31st, 2021 were retrospectively collected. This pediatric ward is placed in a general hospital without PICU.

**Fig. 1:**
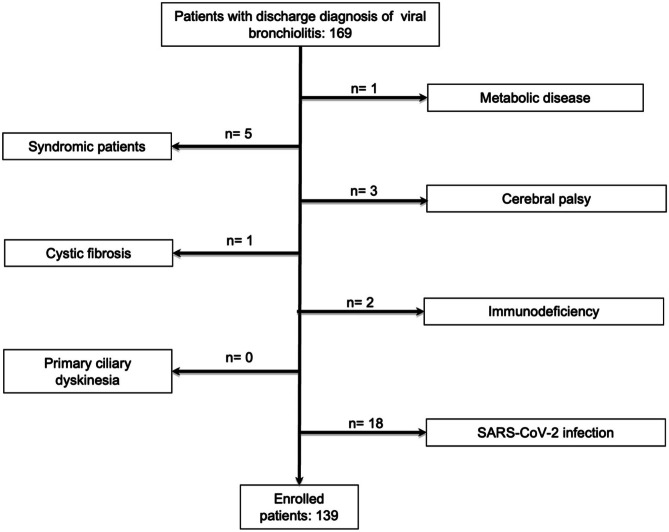
Flowchart describing patients’ enrolment

Inclusion criteria were as follows: (i) age between 1 and 12 months of life; (ii) discharge diagnosis of viral bronchiolitis; and (iii) availability of serum creatinine levels at admission. We excluded patients with known metabolic disease (*n* = 1), syndromes (*n* = 5), cerebral palsy (*n* = 3), cystic fibrosis (*n* = 1), immunodeficiency (*n* = 2), and primary ciliary dyskinesia (*n* = 0) potentially affecting the severity of the viral bronchiolitis (Fig. 1). Moreover, since the outbreak of SARS-CoV-2 pandemic, all the patients at admission also underwent to a molecular SARS-CoV-2 nasal swab. All the patients that met the inclusion criteria but presented with SARS-CoV-2 infection (*n* = 18) were excluded from the study (Fig. 1). This was because patients with SARS-CoV-2 infection were centralized—as per local organization—in a different hospital. Furthermore, all the enrolled patients underwent to nasal swab for the FILMARRAY^®^ Respiratory Panel multiplex polymerase chain reaction. Serum creatinine measurement at the time of admission was available for all the enrolled patients. The study was approved by the Research Ethical Committee of University of Campania (approval no. 12770/2020). Before any procedure, an informed consent was obtained.

### Data collection

The collected data are shown in Table 1. All the available serum creatinine values were recorded. In 138 out of 139 (99.3%) patients, a second serum creatinine assessment in addition to the baseline measurement was available. A second serum creatinine measurement was available in all the patients with AKI. Serum creatinine was measured in 89 out of 138 patients after 24 h of stay (64.5%), in 30 (21.7%) after 48 h, in 6 (4.4%) after 72 h, and in 13 (9.4%) after 96–144 h.

**Table 1:** Clinical and laboratory characteristics of all enrolled patients, and of those with and without AKI

		**All patients** **No. = 139**	**AKI (no)** **No. = 124**	**AKI (yes)** **No. = 15**	***p***
Demographic and clinical features	Age, months, mean (SDS)	3.2 (2.1)	3.1 (2.1)	3.8 (1.1)	0.24
	Male gender, no. (%)	82 (58.9)	72 (51.8)	10 (66.7)	0.52
	Birth weight, kg, mean (SDS)	3.0 (0.6)	3.1 (0.6)	2.6 (0.7)	0.003
	Birth weight < 10th percentile, no. (%)	19 (13.7)	13 (10.5)	6 (40.0)	< 0.001
	Preterm birth, no. (%)	29 (10.9)	20 (16.1)	9 (60)	0.001
	Gestational age, weeks, median (IQR)	38.0 (2.2)	38.2 (2.0)	36.5 (2.7)	< 0.001
	Duration of symptoms before admission, days, mean (SDS)	3.7 (2.5)	3.7 (2.5)	4.1 (2.5)	0.57
	Weight, percentiles, mean (SDS)	40.7 (31.9)	41.3 (30.8)	36.3 (40.6)	0.56
	Signs of respiratory distress, no. (%)	118 (84.9)	106 (85.5)	12 (80.0)	0.57
	Respiratory rate > 2SDS, no. (%)	9 (6.5)	8 (6.4)	1 (6.7)	0.97
	Presence of fever, no. (%)	31 (22.3)	30 (24.2)	1 (6.7)	0.12
	Refill > 2 s, no. (%)	0 (0)	0 (0)	0 (0)	0.99
	HR > 2SDS for age, no. (%)	0 (0)	0 (0)	0 (0)	0.99
	Glasgow Coma Scale < 15, No. (%)	0 (0)	0 (0)	0 (0)	0.99
	Saturimetry at admission, %, median (IQR)	96 (2.0)	96 (2.0)	97 (2.0)	0.62
	Length of stay, days, median (IQR)	5.0 (3.0)	4.0 (3.0)	5.0 (2.0)	0.35
Treatments received	Need of oxygen therapy, no. (%)	118 (84.9)	107 (86.3)	11 (73.3)	0.18
	Need of high flow oxygen therapy, no. (%)	3 (2.1)	3 (2.4)	0 (0)	0.54
	Need of intubation, no. (%)	0 (0)	0 (0)	0 (0)	0.99
	Use of nephrotoxic drugs, no. (%)	2 (1.4)	2 (1.6)	0 (0)	0.83
	Need of intravenous rehydration*, no. (%)	28 (20.1)	25 (20.2)	3 (20.0)	0.99
	Need of bolus, no. (%)	0 (0)	0 (0)	0 (0)	0.99
Biochemical results	WBC, n/mcL, median (IQR)	10,065 (5750)	10,020 (5680)	11,310 (6880)	0.52
	Neutrophils, n/mcL, median (IQR)	3450 (3140)	3450 (3070)	3450 (4100)	0.93
	Serum creatinine levels at admission, mg/dL, median (IQR)	0.3 (0.1)	0.2 (0.1)	0.6 (0.1)	< 0.001
	eGFR at admission, mL/min/1.73 m2, median (IQR)	91.7 (44.3)	116.1 (40.4)	41.6 (11.6)	< 0.001
	Serum urea levels, mg/dL, mean (SDS)	9.0 (3.1)	9.1 (3.1)	8.9 (2.7)	0.88
	Urinary specific gravity^**^, median (IQR)	1010 (10)	1010 (10)	1011 (4.0)	0.30
	Serum Na level, mEq/L, mean (SDS)	137.4 (3.3)	137.5 (2.9)	136.8 (5.9)	0.45
	Serum Na levels > 145 mEq/L, no. (%)	1 (0.7)	0 (0)	1 (6.7)	0.10
	Serum Na levels < 135 mEq/L, no. (%)	24 (17.2)	18 (14.5)	6 (40.0)	0.01
	Serum chloride levels, mEq/L, mean (SDS)	100.9 (3.1)	100.7 (3.1)	102.6 (3.3)	0.04
	Serum potassium levels, mEq/L, mean (SDS)	5.4 (0.84)	5.4 (0.84)	5.5 (0.89)	0.92
	Serum phosphates levels, mg/dL	6.0 (0.64)	6.0 (0.6)	5.6 (0.65)	0.06
	Serum calcium levels, mg/dL	10.3 (0.49)	10.3 (0.49)	10.3 (0.35)	0.63
	Hematocrit > 2SDS, no. (%)	18 (12.9)	12 (9.9)	6 (40.0)	0.001
	Hematocrit < 2SDS, no. (%)	21 (15.1)	21 (17.4)	0 (0)	0.08
	C-reactive protein, mg/dL, mean (SDS)	0.65 (1.1)	0.66 (1.1)	0.51 (1.2)	0.62
	Procalcitonin, ng/mL, mean (SDS)	0.41 (1.0)	0.38 (0.6)	0.53 (0.8)	0.76
	RSV infection, no. (%)	74 (53.2)	61 (49.2)	13 (86.74)	0.006

For normal distributed variables, means ± SDS are shown, while for non-parametric ones, median and interquartile range are shown

*AKI* acute kidney injury, *eGFR* estimated glomerular filtration rate, *HR* hearth rate, *IQR* interquartile range, *Na* sodium, *RSV* respiratory syncytial virus, *SDS* standard deviation score, *WBC* white blood cell count

^*^In case of need of intravenous rehydration, the patients underwent normal saline infusion

^**^Available in 66 patients, 60 without and 6 with AKI

### Case definition

Isotope dilution mass spectrometry-traceable method was used to measure serum creatinine levels.

AKI was defined by the serum creatinine criterion indicated by the Kidney Disease/Improving Global Outcomes (KDIGO) [12]. We estimated the basal serum creatinine value by previously validated back-calculation methods [13]. Since the height of patients was often missing, we used the Hoste (age) equation to calculate the estimated glomerular filtration rate (eGFR) and to back-calculate basal serum creatinine [14], assuming that the basal eGFR represented the median age-based eGFR normative values [15]. The current literature indicates a similar performance for the height-dependent and age-dependent estimation methods of the basal serum creatinine [13].

In babies with preterm birth, we used the corrected age to calculate the eGFR and to estimate basal serum creatinine [16].

Creatinine values < 1.5, 1.5 to < 2, 2 to < 3, and ≥ 3 times the basal creatinine defined respectively no AKI, stage 1, stage 2, and stage 3 AKI [12].

The KDIGO urine output criterion was not considered since this data was not recorded in the clinical charts.

### Other definitions

Viral bronchiolitis was defined as the first episode of wheezing in infants younger than 12 months of age [1].

Heart rate (HR) and respiratory rate (RR) higher than 2 standard deviation score (SDS) based on the percentiles for age and body temperature defined respectively increased HR and RR [17, 18].

A Glasgow coma scale < 15 was used to identify patients with consciousness impairment.

Hematocrit levels were defined on the basis of age-specific percentiles [19].

Fever was defined by axillar body temperature > 38 °C.

Preterm birth was defined by birth completed before 37 weeks of pregnancy [20].

Infants with birth weight < 10th percentile were defined as small for gestational age (SGA) [21].

### Post-hoc power calculation

A previous study reported an AKI prevalence of 4.5% in infants affected by viral bronchiolitis in a non-PICU setting [8]. Considering this prevalence and the one of our study (10.8% in the 139 subjects with viral bronchiolitis), the post hoc power, with an alpha of 0.05, was of 86.1%.

### Statistical analysis

We considered statistically significant *p* value < 0.05. When analyzing continuous variables, we used the independent-sample *t* test in case of normal distribution and the Mann–Whitney test in case of non-normal distribution. The normality of continuous variables was evaluated by skewness and kurtosis statistics*.* The chi-square text or —when appropriate— the Fisher exact test was used to compare qualitative variables. To explore the association with AKI, we used logistic regression models. The variables associated with AKI (*p* < 0.05), when compared to the features of the patients with and without AKI (Table 1), were added in the univariate logistic regression analysis. In turn, the variables with *p* < 0.05 at univariate analysis were included in the multivariate analysis. At multivariate analysis, only the variables with a significant *p* after Bonferroni correction were considered statistically significant. Considering that 5 variables were added in the multivariate analysis, the *p* was significant if less than 0.01.

The Stat-Graph XVII and SPSS 25 software for Windows were used for the statistical analyses of this manuscript.

## Results

One hundred and thirty-nine patients (41.1% of female gender) with mean age 3.2 ± 2.1 months (range: 1–11 months) were enrolled (Fig. 1). None of the enrolled patients required mechanical ventilation or transport in PICU. All the patients presented a normal prenatal ultrasound and none of them presented known nephro-uropathies. RSV infection was detected in 74 out of 139 patients (53.2%) (Table 1). Among the 74 patients with RSV infection, 4 presented a coinfection with Rhinovirus. In 15 (10.8%) patients only human Metapneumovirus, in 19 (13.7%) only Rhinovirus, in 8 (5.8%) only Enterovirus, in 16 (11.5%) only Parainfluenza viruses 1–4, and in 3 (2.1%) only Adenovirus were detected. In 4 (2.9%) patients, the infectious agent remained unidentified. The clinical features of our population are shown in Table 1.

Out of 139 patients, AKI was found in 15 (10.8%). AKI was found in 13 out of 74 (17.6%) patients with and in 2 out of 65 (3.1%) without RSV infection (*p* = 0.006).

No patient required renal replacement therapies, 1 out of 15 (6.7%) developed AKI stage 3, 1 (6.7%) developed AKI stage 2, and 13 (86.6%) developed AKI stage 1. Both patients reaching AKI stage 3 and stage 2 had RSV infection. One patient with RSV and Rhinovirus coinfection presented with AKI stage 1.

Among the 15 patients with AKI, 13 (86.6%) reached the highest AKI stage at admission, 1 (6.7%) at 48 h, and 1 (6.7%) at 96 h.

Patients with AKI presented lower gestational age and birth weight and higher prevalence of preterm birth, birth weight < 10th percentile (small for gestational age), serum Na levels < 135 mEq/L, hematocrit > 2SDS, and RSV infection than patients without AKI (Table 1). As expected, patients with AKI presented higher serum creatinine and lower eGFR levels compared to those without AKI (Table 1).

At exploratory univariate logistic regression analysis of factors potentially associated with AKI, birth weight < 10th percentile, preterm birth, RSV infection, serum Na levels < 135 mEq/L, and hematocrit levels > 2SDS were significant and then included in the multivariate analysis (Table 2). At multivariate analysis, birth weight < 10th percentile, preterm birth, RSV infection, and hematocrit levels > 2SDS persisted significantly associated with AKI after Bonferroni correction.

**Table 2:** Exploratory analysis of factor potentially associated with AKI

	**Univariate analysis**^**b**^			**Multivariate analysis**^**c**^		
	**OR**	**95% CI**	***p***	**OR**	**95% CI**	***p***
Birth weight < 10th percentile	5.7	1.7/18.6	0.004	34.1	3.6/324.9	0.002
Preterm birth	7.8	2.5/24.3	< 0.001	20.3	3.1/129.5	0.002
RSV infection	6.7	1.4/30.9	0.01	27.0	2.6/279.9	0.006
Serum Na levels < 135 mEq/L	3.9	1.2/12.4	0.02	6.2	0.8/44.7	0.07
Serum chloride levels^a^	1.2	1.0/1.5	0.05	-	-	-
Hematocrit > 2SDS	6.0	1.8/19.9	0.003	22.4	2.8/183.6	0.004

*AKI* acute kidney injury, *Na* sodium, *RSV* respiratory syncytial virus, *SDS* standard deviation score

^a^1 mEq/L increase in chloride levels

^b^We included in the multivariate analysis only the variables with *p* < 0.05 at univariate analysis

^c^We considered significant at multivariate analysis only the variables with significant *p* after Bonferroni correction. The significant *p* value after Bonferroni correction was < 0.01

## Discussion

Despite the non-PICU setting of the study, our data confirmed that a considerable percentage (about 11%) of patients with viral bronchiolitis may develop AKI. This prevalence is higher than the one (7.5%) reported by Angurana et al. [8], which —unlikely— did not provide the methods to diagnose AKI nor the different stages of the disease. Probably, only the more severe AKI cases were accounted in such paper [8].

Considering that 47,000–172,000 children are yearly hospitalized for viral bronchiolitis [2, 3] and that 11% of them will develop AKI (as found in our study), in the USA, 5,000–19,000 children with viral bronchiolitis could yearly develop AKI.

During the hospitalization, none of our patients was diagnosed with AKI. This, in addition to the presence of only one report describing an association between AKI and bronchiolitis [8], further confirms our meaning that AKI, especially in its milder forms, is often under-recognized in children [4–7].

While the effect of this under-diagnosis seems to be irrelevant in the short term as the discharge after a mean length of stay of only about 5 days for all patients, AKI might significantly affect patients’ health by increasing the risk of future CKD [11]. In fact, even if AKI is usually reversible on the basis of serum creatinine concentrations, the persistence of a subclinical kidney damage (e.g., kidney fibrosis) might predispose to CKD development in the following years [11, 22–24]. Mammen et al. showed that 10% of 126 critically ill children with AKI and no pre-existing CKD developed CKD after 1–3 years of follow-up [25]. The separate analysis of patients with AKI stage 1 showed a CKD prevalence of 4.5% at 1–3 years of follow-up [25]. To date, no prognostic evaluations in children who developed AKI without the need of PICU admission have been performed. Waiting for more evidence, being able to identify patients with AKI (also in its milder forms) could be relevant to set up a specific follow-up with the aim to early identify signs of CKD and to counteract its progression (i.e., good blood pressure control and antiproteinuric drugs) [26].

Preterm birth and birth weight less than 10th percentile were significantly associated with AKI development. This might be related to the fact that both conditions are associated with a reduced nephronic mass [27, 28]. More, hematocrit > 2SDS was closely associated with AKI, suggesting a role of dehydration in the pathophysiology of AKI in viral bronchiolitis. Since most patients already developed AKI on admission, however, there may be limitations in using laboratory parameters such as hematocrit levels to change the clinical practice and to prevent AKI or improve outcomes.

Interestingly, RSV was significantly associated with AKI development and persisted significantly also after multivariate analysis, adding further evidence about the extrapulmonary manifestation of RSV in young children [1]. Evidence indicates that RSV is able to determine several extrapulmonary manifestations affecting cardiovascular (i.e., myocarditis or arrhythmias), central nervous (i.e., apneas), endocrine (i.e., increased antidiuretic hormone levels), and gastrointestinal systems (i.e., hepatitis) [29]. These various conditions suggest that RSV may have an impact behind the lung [29]. However, indirect extrapulmonary effects of the RSV might also occur [29], likely due to an abnormal inflammatory response of the host to RSV [30, 31]. Considering that severe systemic inflammation could determine AKI (as in the case of community-acquired pneumonia [5] and SARS-CoV-2 infection [32]), there is a chance that the systemic inflammation could be the link between RSV and AKI.

Our study group previously analyzed both prevalence of and factors associated with AKI in different groups of children [4–7]. In children with acute gastroenteritis, we found lower birth weight and higher hematocrit levels in patients who developed AKI compared with those who did not [6], while in children with community-acquired pneumonia, we found lower birth weight and higher percentage of preterm birth in patients developing AKI compared with those who did not [5]. In both cases, however, these parameters did not persist significantly at multivariate logistic regression analyses. This could be probably related to the fact that every illness has mainly specific pathophysiological mechanisms in the AKI development (i.e., dehydration in acute gastroenteritis and systemic inflammation in community-acquired pneumonia) [5, 6]. Since viral bronchiolitis generally affects infants [1], in this case, the effect of preterm birth and low birth weight on the renal function could be more evident than in the other illnesses usually occurring in older children and with different pathophysiology [5, 6].

Evidence from children with type 1 diabetes mellitus shows AKI resolution in all children within 14 days [7]. Therefore, it is reasonable to hypothesize that also in the case of viral bronchiolitis, in which we observed milder AKI forms than in type 1 diabetes mellitus [7], AKI could be completely reversible. However, even if specific studies are lacking, there is a chance that —even in the case of viral bronchiolitis— AKI could determine a subclinical kidney damage predisposing to the future CKD development.

Limitations of our study are represented by (i) the retrospective design; (ii) unavailability of measured basal serum creatinine, in any case difficult to obtain also in future longitudinal studies as in the first months of life children do not usually undergo routine biochemical exams; (iii) lack of data about urinary output which prevent us the use of the urine output KDIGO criterion to diagnose AKI with a possible underestimation of the AKI prevalence; (iv) lack of information in clinical charts about palivizumab immunoprophylaxis in preterm patients that did not allow us to evaluate a possible protective effect against AKI of this immunoprophylaxis; and (v) lack of follow-up data of these patients that do not allow us to investigate the percentage of complete AKI resolution.

In conclusion, we showed that about 11% of patients hospitalized with viral bronchiolitis in a non-PICU setting can develop AKI (frequently mild in degree). Preterm birth, birth weight < 10th percentile, hematocrit levels > 2 SDS, and respiratory syncytial virus infection were associated with AKI development. Our data suggests that in children with viral bronchiolitis, the reduced nephronic mass (as in the case of preterm or SGA babies), the dehydration (with hematocrit > 2SDS as biomarker), and the RSV infection play an important role.

## Data Availability

The dataset generated during and/or analyzed during the current study are available from the corresponding author on reasonable request.

## Abbreviations

AKI: Acute kidney injury
CAKUT: Congenital anomalies of kidney and urinary tract
CKD: Chronic kidney disease
eGFR: Estimated glomerular filtration rate
HR: Heart rate
KDIGO: Kidney Disease/Improving Global Outcomes
OR: Odds ratio
PICU: Pediatric intensive care unit
RR: Respiratory rate
RSV: Respiratory syncytial virus
SDS: Standard deviation score
SGA: Small for gestational age

## Code availability

N/A.
